# The FCG Editor: An innovative environment for engineering computational construction grammars

**DOI:** 10.1371/journal.pone.0269708

**Published:** 2022-06-09

**Authors:** Remi van Trijp, Katrien Beuls, Paul Van Eecke

**Affiliations:** 1 Sony Computer Science Laboratories Paris, Paris, France; 2 Faculté d’informatique, Université de Namur, Namur, Belgium; 3 Artificial Intelligence Laboratory, Vrije Universiteit Brussel, Brussels, Belgium; 4 Itec, Imec Research Group at KU Leuven, Kortrijk, Belgium; University of California, San Diego, UNITED STATES

## Abstract

Since its inception in the mid-eighties, the field of construction grammar has been steadily growing and constructionist approaches to language have by now become a mainstream paradigm for linguistic research. While the construction grammar community has traditionally focused on theoretical, experimental and corpus-based research, the importance of computational methodologies is now rapidly increasing. This movement has led to the establishment of a number of exploratory computational construction grammar formalisms, which facilitate the implementation of construction grammars, as well as their use for language processing purposes. Yet, implementing large grammars using these formalisms still remains a challenging task, partly due to a lack of powerful and user-friendly tools for computational construction grammar engineering. In order to overcome this obstacle, this paper introduces the FCG Editor, a dedicated and innovative integrated development environment for the Fluid Construction Grammar formalism. Offering a straightforward installation and a user-friendly, interactive interface, the FCG Editor is an accessible, yet powerful tool for construction grammarians who wish to operationalise their construction grammar insights and analyses in order to computationally verify them, corroborate them with corpus data, or integrate them in language technology applications.

## Introduction

Interest in constructionist approaches to language, as pioneered by among others Fillmore [1], Goldberg [2], Kay & Fillmore [3] and Croft [4], has been steadily growing over the last four decades. In the meantime, the key foundational ideas underlying construction grammar have been successfully adopted in many subfields of linguistics, including language acquisition [5, 6], psycholinguistics [7], language learning and teaching [8, 9], historical linguistics [10] and language evolution and change [11, 12].

While the methodological focus of the construction grammar community has traditionally been on theoretical [13], corpus-based [14, 15] and experimental research [16, 17], the importance of computational methodologies is now rapidly increasing. This movement is witnessed by the increased presence of talks, tutorials and courses on computational construction grammar at international conferences and schools, such as the 2014 Language Resources and Evaluation Conference (LREC), the 2017 Interdisciplinary College (IK) Spring School, the 2020 LOT Winter School, and the 2018 and 2021 International Conferences on Construction Grammar (ICCG). Computational approaches bear the promise of establishing more solid foundations for the field of construction grammar and of expanding its application potential, as they make it possible to (i) automatically verify the precision and internal consistency of linguistic analyses [18–24], (ii) corroborate these analyses with corpus data [20, 25–28], and (iii) exploit linguistic insights and analyses for enhancing the performance of language technology applications [29–32].

Catering to these needs, a number of formalisations and computational implementations of construction grammar have seen the light of day, each with their own goals and methodologies. The three most influential efforts have been Embodied Construction Grammar (ECG) [33, 34], Fluid Construction Grammar (FCG) [35, 36] and Sign-Based Construction Grammar (SBCG) [37, 38]. Each of these provides at least a formalism in which constructions can be specified, as well as unification-based algorithms that can use these constructions for comprehending (ECG, FCG, SBCG) or formulating (FCG, SBCG) natural language utterances. ECG and FCG come with their own computational implementations, while SBCG grammars could in principle be processed using existing systems for implementing typed feature structure grammars, for example LKB [39] or TRALE [40] (cf. the debate between van Trijp [41] and Müller [42] on the faithfulness of such implementations).

Yet, despite the availability of these formalisations and their computational implementations, it remains a challenging task to operationalise construction grammars of considerable size. This is partly due to a lack of user-friendly environments for writing, testing, inspecting and debugging constructions. Without such tools, it is tremendously difficult to keep track of the intricate relations between the constructions of a grammar, very much like it is difficult to write large computer programs using a basic text editor only.

In this paper, we aim to remedy this situation by introducing the FCG Editor, an innovative integrated development environment (IDE) for the Fluid Construction Grammar formalism. The FCG Editor offers a straightforward installation and a user-friendly interface, which enables users to write, visualise, process and debug their construction grammars. The FCG Editor aims to strike a unique balance between user-friendliness and open-endedness, drawing inspiration from the field of interactive programming. In this spirit, users can track in detail all interactions between constructions during language processing, and add, delete or modify constructions or processing configurations on the fly.

The target user group of the FCG Editor are linguists, in particular construction grammarians, who wish to computationally operationalise their construction grammar insights and analyses. Computational operationalisations come with four main advantages. First of all, they validate the internal consistency of an analysis, as they immediately reveal any inconsistencies or errors. Second, they make it possible to corroborate construction grammar theories with large amounts of corpus data, unequivocally showing what they can and cannot account for. Third, they help to standardise how constructions are represented and processed, facilitating the comparison, exchange and integration of contributions by different researchers. Finally, they make it possible to exploit construction grammar insights and analyses for enhancing the performance of language technology applications.

## Background and related work

### Constructionist approaches to language

Over the last four decades, constructionist approaches to language have been gaining increasingly more attention in the linguistic community and have by now become a mainstream paradigm for linguistic research. The phrase *constructionist approaches to language*, as coined by Goldberg [43], refers to a family of linguistic theories which share a number of key foundational principles. Based on the work of the main architects of construction grammar (cf. [2–4, 44, 45]), we distinguish the following basic tenets:

1. **All linguistic knowledge is captured in the form of constructions**. Constructions (cxns for short) are defined as form-meaning pairings that facilitate the comprehension and production of linguistic utterances. Comprehension corresponds to the process of mapping from an utterance to its meaning representation, while production corresponds to the inverse process of mapping from a meaning representation to an utterance that expresses it.
2. **There exists a lexicon-grammar continuum, with no distinction between “words” and “grammar rules”**. Each construction is situated somewhere on this continuum. Constructions can range from entirely idiomatic expressions, over partially productive patterns, to entirely abstract schemata. Examples of these types of constructions are respectively (i) the break-a-leg-cxn, which constitutes a holistic pairing between the utterance “*break a leg!*” and the meaning of wishing an addressee good luck, (ii) the x-take-y-for-granted-cxn, which includes variable slots for the agent and the undergoer, and expresses that the former does not value the latter, and (iii) the resultative-cxn in “*the Tasmanian tiger was hunted to extinction*”, which expresses that the Tasmanian tiger was extinct as a result of hunting.
3. **Constructions can contain information from all levels of linguistic analysis**. Construction grammar does not make an a priori distinction between the different layers of traditional linguistic analysis, such as phonetics, phonology, morphology, syntax, semantics and pragmatics. Constructions can, but do not need to, include information from any of these layers at the same time, as long as they constitute a mapping between some aspects of meaning and some aspects of form. It is entirely open what the form side and the meaning side of a construction can contain. For example, the form side typically includes phonetic, phonological, morphological, syntactic or multimodal information, while the meaning side typically includes semantic or pragmatic information.
4. **Construction grammars are dynamic systems, of which the constructions and their entrenchment are in constant flux**. Constructions always represent the linguistic knowledge of an individual language user. Constructions are acquired and change over time. They can be more or less entrenched as they are used more or less frequently and successfully.

As is normal for a young scientific discipline, the exciting new ideas underlying construction grammar were not immediately precisely defined, let alone formalised or computationally operationalised. The initial grand ideas needed to settle first, before more solid foundations could be established. However, as the discipline has matured, sound foundations, formalisations and computational operationalisations are now an essential part of the construction grammar enterprise. Investigating the nature of these foundations gave rise to an entire new subfield of construction grammar, called *computational construction grammar*.

### Computational construction grammar

The field of computational construction grammar explicitly aims to provide precise formalisations of the building blocks of construction grammar, as well as fully operational processing models [23]. These formalisations and processing models are important from both a theoretical and a practical perspective. On the theoretical side, they are a crucial instrument supporting the assessment of the consistency and coverage of construction grammar analyses. On the practical side, they facilitate the use of construction grammar insights and analyses in language technology applications, such as visual question answering systems [30], the frame-semantic analysis of discourse [31, 32] and tools for exploring large corpora from a construction grammar perspective [46].

Since the early 2000s, a number of formalisations and computational operationalisations of construction grammar have emerged, each approaching the challenge from a different perspective:

- **Sign-Based Construction Grammar (SBCG)** [37] builds further on earlier work in the generative linguistics tradition, adopting the formal machinery and theoretical foundations of Head-Driven Phrase Structure Grammar (HPSG) [47, 48]. In particular, SBCG extends HPSG’s typed feature structure-based backbone with a distinction between signs and constructs, so that idiosyncratic phenomena and syntactico-semantic constraints affecting larger patterns can more elegantly be handled [37, 49]. SBCG incorporates in this way a foundational principle from construction grammar, while remaining at the same time deeply rooted in the generative and phrase structure grammar traditions. Like HPSG and other phrase structure grammars, SBCG adheres to a dictionary-and-grammar constellation [50] as opposed to a lexicon-grammar continuum, enforces locality on grammar rules [51], and does not aim to model grammars as dynamically emerging and evolving as a result of their use in communication [43, 52]. As SBCG grammars adopt the same formal machinery and theoretical foundations as HPSG grammars, they could in principle be processed using the same computational tools, in particular LKB [39] and TRALE [40].
- **Embodied Construction Grammar (ECG)** [33, 34] is a computational construction grammar implementation that provides a formalism and unification-based algorithms for operationalising constructional language processing in the comprehension direction. Starting from the constructionist assumptions that the basic units of linguistic knowledge are pairings between form and meaning, i.e. constructions, and that language serves to convey meaning using form, research in the ECG tradition focusses on the cognitive and neural mechanisms involved in language use [53], in particular on the representation of meaning as schemata that are embodied in the human sensory-motor system [54].
- **Fluid Construction Grammar (FCG)** [36, 55] is a computational implementation of the basic tenets of construction grammar. It can be seen as a special-purpose programming language for computationally operationalising construction grammar insights and analyses. It provides a construction grammar formalism, a unification engine supporting constructional language processing in both the comprehension and production direction, and an extensive library of building blocks that can readily be used by the construction grammar engineer. These building blocks include data structures and algorithms for operationalising constructions, construction inventories, heuristic search processes, meta-level learning operators, entrenchment processes, and networks of grammatical categories. FCG strives to be a flexible system for exploring novel construction grammar ideas. Apart from adhering to the basic tenets of construction grammar, it thereby imposes as few theoretical assumptions as possible.
- **Dynamic Construction Grammar (DCG)** [56] and **Template Construction Grammar (TCG)** [57] are neuro-computational approaches to construction grammar. DCG makes use of artificial neural networks to find regularities in the mappings between utterances and their argument structure. TCG was developed to investigate how language and vision processing interact on a neural level in the human brain. Both DCG and TCG are experimental approaches which at this point do not provide computational tools for end-users.

The substantive body of research that has by now been yielded by the field of computational construction grammar has not only helped to establish more solid foundations for the constructionist view on language, but has in the meantime also resulted in a number of impactful real-world applications [30–32, 46, 58–61]. Yet, the grammars that are currently available are either fragments targeted towards detailed analyses of specific linguistic phenomena of interest, including the English auxiliary system [62, 63], English measure phrases [64], English caused-motion constructions [23, 65], English long-distance dependencies, [42, 66], English metaphors [67], Dutch modal stacking [68], Hungarian poly-personal agreement [69] and tense, aspect and modality in the Spanish verbal system [70], or application-specific grammars that were designed for optimal performance on a predefined task [30, 32]. Some attempts have been made to create large, domain-general, fine-grained computational construction grammars, either by leveraging FrameNet data to expand the coverage of seed grammars [71, 72], or by combining a set of fully instantiated constructions that were automatically created based on lexical resources with a collection of hand-crafted constructions that handle more abstract patterns [73]. While all of these attempts contributed interesting ideas and operational models, they showed at the same time that the success of large-scale, broad-coverage construction grammar engineering crucially hinges on the computational tools that are available to the grammar engineer. This insight was of course not new, nor is the issue proper to construction grammar engineering. Indeed, research on grammar engineering in the generative linguistics tradition has elaborately studied many aspects relating to this issue, and has proposed a variety of solutions in the form of both theoretical insights and fully-operational tools.

### Construction grammar engineering: Issues and tools

Much of the foundational work on large-scale, broad-coverage grammar engineering was carried out in the 1980s and 1990s by researchers trained in a variety of computational linguistics formalisms, in particular Tree-Adjoining Grammar (TAG) [74], Lexical-Functional Grammar (LFG) [75], Combinatory Categorial Grammar (CCG) [76] and Head-Driven Phrase Structure Grammar (HPSG) [77]. Since this period, many research papers have been published that explicitly tackle issues related to large-scale grammar engineering or that present tools for supporting the grammar engineering process. These issues involve the automatic verification of the coverage and precision of a grammar, including approaches to regression testing and writing maintainable grammars [78–83], the coordination between researchers in collaborative grammar engineering [84, 85], and grammar debugging [78, 86]. A wide range of tools have been developed to support grammar engineering using these formalisms, in particular TuLiPA [87] for TAG, XLE [88, 89] for LFG, DotCCG [90] and GF [91] for CCG, ALE [92], TRALE [40] and LKB [39] for HPSG, and Hdrug [93] for a variety of formalisms, including flavours of HPSG, TAG and CCG. These tools have not only made it possible to implement grammars of considerable size for a large number of languages, but have also facilitated the exchange of grammars between researchers in the computational linguistics community. For a comprehensive overview of available grammars implemented in these formalisms, we refer the reader to [94].

Most solutions that have resulted from research on grammar engineering in the generative grammar tradition can directly be reused for construction grammar engineering, as many challenges, including verification, collaborative grammar writing and grammar debugging, remain the same. Other engineering challenges are specific to construction grammars and therefore require bespoke solutions. First of all, the construction-based comprehension and production of linguistic utterances relies on the free combination of constructions as long as no conflicts occur [23, 45]. Combined with the non-locality of constructions, the fact that constructions do not need to include word order constraints [4] and that they do not necessarily correspond to tree-building operations [95], this means that constructional language processing cannot faithfully be implemented using well-known optimisation techniques, such as chart parsing and generation [96, 97]. Moreover, it is quite common that many different combinations of construction applications can lead to a valid solution and that possible variations are at least partly motivated by entrenchment phenomena. The construction application process is usually implemented as a rather expensive search problem [98], and large-scale construction grammar engineering can, at least today, not completely be decoupled from optimization techniques. It is therefore of crucial importance for the construction grammar engineer to be able to inspect in detail the search process involved in constructional language processing, and to have easy access to optimisation solutions such as the scoring and hashing of constructions, the use of heuristics and priming methods [99, 100], and the efficient organisation of the constructions in the construction inventory [69]. Another challenge specific to construction grammar engineering stems from the nature of the underlying linguistic theory. Constructionist approaches to language, by definition, adhere to the basic tenets of construction grammar discussed above, but many other aspects, including the nature of grammatical categories, family relations between constructions, and the integration of ontological knowledge and common sense reasoning into constructional language processing, are still very much in an exploratory stage. This requires systems and tools for construction grammar engineering to be flexible, open-ended and extensible, so that novel ideas can easily be explored. On the one hand, this open-endedness ensures that construction grammarians do not feel constrained by the computational tools they use. On the other hand, it makes sure that computational research in construction grammar can directly contribute to theory building.

There exist today two main software platforms that aim to provide a faithful operationalisation of the basic tenets of construction grammar and as a consequence need to address the challenges specific to constructional language processing discussed above. These are the ECG workbench for Embodied Construction Grammar [101, 102] and the Babel platform for Fluid Construction Grammar [86, 103]:

- The **ECG workbench** is an integrated development environment that allows construction grammar engineers to create and explore their own grammar fragments, and use them for comprehending individual utterances. Apart from an editing window where construction definitions can be displayed and altered, the workbench also hosts the functionality to visualise the end result of a constructional analysis in the form of a semantic specification, called *SemSpec*. The SemSpec “specifies the conceptual schemas evoked by the constructions involved [in an analysis] and how they are related” [33]. While the end result of a construction application process can be inspected in a graphical way, the process itself is kept hidden from the user. Only the names and scope of the constructions and schemas that contributed to the resulting semantic specification are returned, together with a number that indicates the cost of the analysis. Interactivity in the development environment is limited to entering utterances, and starting or interrupting the comprehension process. The ECG workbench does not include the possibility to inspect the construction application process itself, keeping optimization and debugging—and therefore large-scale grammar engineering—a difficult task.
- FCG grammars are designed using the **Babel platform**. Babel is marketed as an all-in-one software library for setting up multi-agent experiments on the emergence and evolution of communication and language [104]. Babel provides building blocks for implementing all aspects involved in such experiments, ranging from the sensory-motor level [105], through the conceptual level [106], to the linguistic level [36]. In essence, Babel is not a computer program or editor, but a software library that includes FCG as a component. FCG is a special-purpose programming language implemented on top of Common Lisp, which provides abstractions for representing and processing construction inventories and constructions in its own syntax. With Babel installed on their machines, FCG users thus directly interact with its source code. They typically make use of general-purpose text editors that implement an interactive Lisp environment, such as Emacs in combination with SLIME. In this environment, users can then write their own grammars and use these in language comprehension and production tasks. FCG comes with elaborate web-based visualisations of all aspects involved in constructional language processing. As Babel is a software library rather than an integrated development environment, it takes a considerable amount of time and effort to become a proficient FCG user as a consequence of the overhead of needing to learn a new programming environment.

Overall, the ECG workbench and the Babel platform have played an important role in the early development of computational construction grammars. However, they have so far not succeeded in adequately addressing the needs of the construction grammar community. The ECG workbench lacks the flexibility that is needed to explore construction grammar ideas that diverge from the initial design choices that were made based on Feldman’s neural theory of language [107], and suffers from significant performance bottlenecks and a lack of interpretability, which make it difficult to design and process larger grammars. The Babel system does not offer an interface that is straightforwardly accessible to construction grammarians and its use requires significant software development skills. Despite the rapidly growing interest in constructionist approaches to language in general, and in computational construction grammar in particular, the community uptake for computational construction grammar implementations has therefore remained limited.

The tool that we present in this paper aims to remedy this situation, and make both large-scale construction grammar engineering and the computational exploration of novel construction grammar ideas accessible to the construction grammarian. Starting from the FCG system, we build a user-friendly, yet flexible, open-ended and extensible tool for construction grammar engineering. The tool is inspired by the best practices in grammar engineering known from the generative grammar tradition, the possibilities offered by the Babel software library, the field of interactive programming, and interactions with target users from within the construction grammar community.

## The FCG Editor

The primary design objective of the FCG Editor is twofold. First of all, the tool should be easily accessible to construction grammarians and support the free exploration of novel construction grammar ideas. Second, the tool should support large-scale, broad-coverage construction grammar engineering, facilitating the fast prototyping of performant computational construction grammars.

This section is subdivided into three parts. First, we report on a requirements analysis that was conducted in collaboration with the construction grammar community. Then, we provide a detailed overview of the design, functionality and main features of the FCG Editor. Finally, we discuss how the system can support both large-scale construction grammar engineering and the computational exploration of new construction grammar ideas.

### Requirements analysis

In order to maximize the potential of the FCG editor in terms of community uptake, a requirements analysis was carried out through discussions with experts in construction grammar and interactions with participants during a number of tutorials on computational construction grammar at international events, including the 2020 LOT Winter School and the 2021 International Conference on Construction Grammar (ICCG). From these extensive discussions, eight criteria that the FCG Editor should satisfy were distilled. While the resulting criteria are rather unsurprising from a software development perspective, they do reflect the concerns that are currently present in the construction grammar community.

The scope of this requirements analysis is bounded to requirements for an integrated development environment for engineering computational construction grammars. A requirements analysis for computational construction grammar formalisms or implementations, like the one provided by Steels [108], explicitly falls outside the scope of this paper.

**R1** The FCG Editor should offer the **basic text formatting functionalities** that are commonly featured in text editors intended for programming. Apart from *open-save-close*, *undo-redo*, *cut-copy-paste* and *find-replace*, these functionalities include adequate syntax highlighting, block comment formatting, auto-indentation, auto-completion and display of function arguments. Key bindings should be intuitive and aligned with those used in other modern editors.
**R2** The editor should offer a **straightforward and cross-platform installation**. External dependencies should be limited to the absolute minimum and they should be freely available. The editor should run on the three major operating systems (Linux, macOS and Microsoft Windows) and be delivered as an executable file that facilitates a single-click installation.
**R3** The editor should aim to make implementing computational construction grammars **more accessible to novice users**, by including tutorials and/or examples which help them to get started with implementing their first constructions.
**R4** The editor should include **visualisations** for constructions and construction application processes, so that they can be graphically inspected by the users. These visualisations should be interactive and allow the users to keep track of, inspect and debug all aspects of constructional processing.
**R5** The editor should include facilities for **catching and displaying insightful error messages**. Debugging information, including system settings, backtraces and memory dumps, should be easily shareable, for example by including the option to print them in the form of plain text reports.
**R6** The editor should allow users to **manage running processes** in an intuitive way. It should be possible to kill a running process, for example when an erroneous construction causes an infinite loop, when a process is no longer responsive, or when constructions create a search space that takes too long to explore.
**R7** The editor should offer the possibility to **write source code that extends the FCG system**. For one thing, it should be possible to extend FCG’s standard library of functions for processing grammars, for example with novel techniques for managing the search process involved in constructional language processing [98, 109]. For another, it should be possible to implement auxiliary functions, for example for automatically creating constructions based on dictionaries, corpora or other linguistic resources, or for applying construction grammars to corpora.
**R8** The editor should provide an **interactive programming environment** through which users can interact with their grammar and the novel code they write. This implies implementing the concept of an active programming session with a Read-Eval-Print-Loop (REPL)-like interface.

In sum, the requirements analysis brought to light three important aspects that have steered the development of the FCG Editor: (i) *user-friendliness* for users who do not have extensive programming experience, (ii) *extensibility* for expert users who wish to develop novel extensions to FCG, and (iii) *interactivity* for all users, so that they can inspect all aspects of constructional language processing through orderly yet detailed visualisations. Other important aspects of construction grammar engineering that have guided the development of the FCG Editor, such as evaluation, regression testing and deployability, did not emerge from the discussions with the community. This can be ascribed to the fact that these aspects are already well covered by the current computational construction grammar implementations, in particular the Fluid Construction Grammar software library [32, 83].

### Design and functionality

The FCG Editor is designed as a user-friendly yet open-ended integrated development environment that facilitates the design and implementation of computational construction grammars using the Fluid Construction Grammar formalism. It is delivered as a stand-alone, executable program that offers a graphical user interface (GUI) through which users can interact with a pre-compiled version of Babel’s FCG software library. In order to maximise the open-endedness of the FCG Editor, its GUI supports the interpretation of both FCG constructs and Common Lisp source code that extends the FCG codebase.

In this section, we first provide an overview of the FCG Editor GUI. We then highlight the main features of the FCG Editor and provide a brief practical guide for new users to get started with the environment. Finally, we share some details about the technical operationalisation of the software.

#### Overview of the FCG Editor GUI

**Fig 1:**
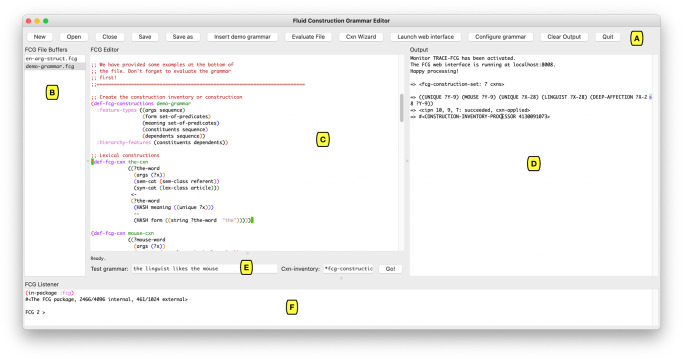
Overview of the graphical user interface. The FCG Editor’s main interface, featuring its toolbar (A), buffer overview list (B), editor pane (C), output pane (D), testing pane (E) and listener pane (F).

When a user opens the FCG Editor, a graphical user interface (GUI) is displayed. The GUI is structured as presented in Fig 1 and consists of six component parts:

A The **toolbar** provides buttons that allow the user to manipulate files (‘New’, ‘Open’, ‘Close’, ‘Save’, ‘Save as’), open an example grammar (‘Insert demo grammar’), create a construction skeleton (‘Cxn Wizard’), evaluate files (‘Evaluate File’), launch the interactive web interface (‘Launch web interface’), launch a graphical grammar configurator (‘Grammar Configurator’), clear all output (‘Clear Output’) and quit the program (‘Quit’).
B The **buffer overview list** provides an overview of all open file buffers, corresponding to a list of all files that are currently open in the FCG Editor. When a file buffer is selected in the buffer overview list, it becomes visible in the editor pane (C).
C The **editor pane**, situated centrally in the GUI, facilitates the editing of both FCG grammars and Common Lisp source code. The editor pane hosts a wide range of functionalities that help users implement their grammars, as will be discussed in more detail below.
D The **output pane** collects all textual output of the FCG Editor, including construction skeletons, grammar configurations, the results of comprehension and production processes, and possible error message reports.
E The **testing pane** allows users to comprehend or produce utterances from the GUI. Users can enter an utterance or a meaning representation, along with the name of a construction inventory. Upon clicking the ‘Go!’ button or hitting Enter, the system processes the utterance (comprehension) or meaning representation (production) using the specified construction inventory.
F The **listener pane** provides a command-line interface to the FCG system. This pane allows the user to interact with the FCG codebase using the Common Lisp programming language. While novice users can safely ignore this pane, it grants expert users the freedom and power to access and/or extend all aspects of the FCG system itself.

Apart from the components discussed above, additional functionality is made available through the menu bar, as well as through dedicated key bindings. The menu bar offers a ‘File’ menu for manipulating files, an ‘Edit’ menu for editing text (including *undo*, *cut-copy-paste* and *find-replace*), an ‘Options’ menu for setting the font and font size of the different panes, an ‘About’ menu providing information about the program and its version, and a ‘Help’ menu that provides a number of manuals and a list of all available key bindings. These include on the one hand the basic ones that are natively provided by the chosen operating system, and on the other hand the standard Emacs key bindings where they do not conflict with native ones.

#### Main features of the FCG Editor

*Advanced text editing*. The editor pane provides advanced text editing functionalities, combining the look-and-feel of modern source code editors with the possibility to use the Emacs-inspired key bindings to which long-time FCG users are used. Basic functionalities, including *open-save-close*, *undo*, *cut-copy-paste* and *find-replace*, are available through the toolbar and menu bar, as well as through their native key bindings. On a more advanced level, the editor pane provides syntax highlighting, auto-completion and auto-indentation functionalities for FCG grammars as well as for Common Lisp source code, improving the readability and ease-of-development of constructs in both syntaxes. Semicolons can be used to indicate single-line comments, while a combination of hashes and pipes can be used to indicate multi-line comments. When hitting the space bar after typing a function or macro call, the expected arguments are displayed immediately below the editor pane.

*Evaluation and execution*. Part of the power of an integrated development environment, as opposed to a plain text editor, lies in its ability not only to support the writing of code, but also its execution. In the case of the FCG Editor, both FCG constructs, such as constructions and construction inventories, and Common Lisp source code can be executed from the editor pane. Execution of FCG constructs consists on the one hand in adding constructions or other linguistic information to the FCG system, and on the other hand in using this information for processing natural language. In line with the functional programming paradigm in which the FCG system is rooted, the FCG Editor refers to execution by the term *evaluation*. Evaluation refers thus to the execution of a block of code by the FCG interpreter, not to the quantitative or qualitative evaluation of a grammar.

From the editor pane, blocks of code written in either FCG or Common Lisp syntax can be evaluated in a number of ways. The most intuitive way for many users is to place the cursor somewhere within the block of code they wish to execute (e.g. a new construction definition) and hit the Shift-Enter key combination. The system then evaluates this block of code (e.g. adding the construction to the construction inventory), writes its output to the output pane if applicable, and puts the cursor in the next block of code. This behaviour mimics the functionality provided by the same key combination in the widely known Jupyter Notebook environment [110]. A second option is to place the cursor immediately after the exact expression to be evaluated and hit the Shift-Ctrl-Enter key combination. This expression is then executed and the output is written to the output pane if applicable. This option mimics a more traditional interactive programming style. Additionally, the button ‘Evaluate File’ from the toolbar can be clicked to evaluate the entire contents of a file. Finally, other Emacs-style key bindings for evaluation, such as Ctrl-x Ctrl-e (on macOS) and Meta-x eval-buffer, are also available.

*Interactive web interface*. The Babel FCG system comes with detailed, interactive visualisations of all aspects involved in constructional language processing. Constructions and construction application processes can be inspected in an orderly yet detailed fashion using an interactive web interface that was especially designed for creating “visualisations for complex data and control structures” [111] through the ubiquitous use of expandable/collapsible elements. The web interface allows users to track the dynamics of the FCG engine during processing, thereby offering an indispensable tool for profiling and debugging complex grammars [112].

**Fig 2:**
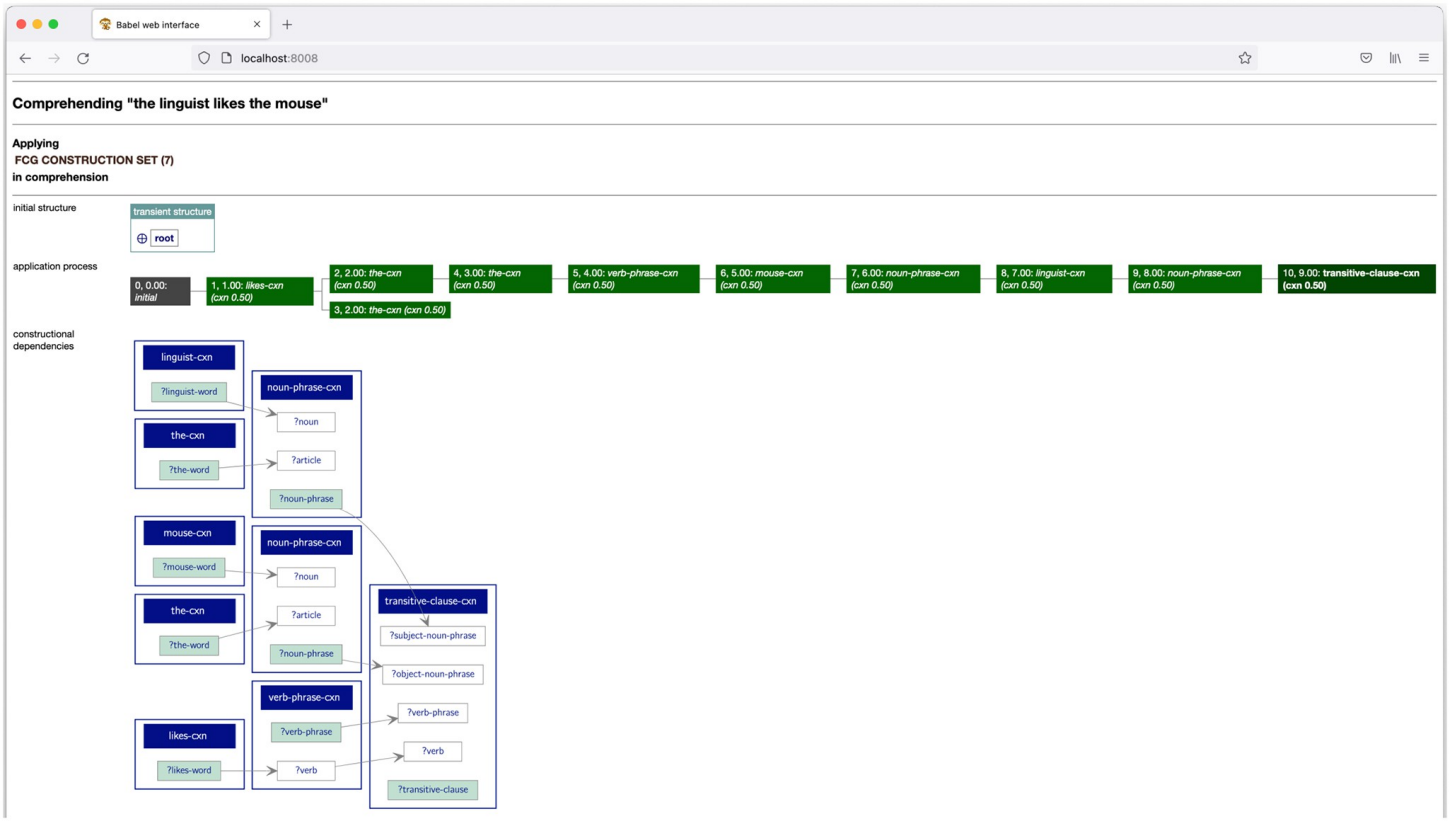
Interactive web interface. The Babel Interactive Web Interface has been integrated into the FCG Editor and is accessible at http://localhost:8008.

The FCG Editor features a complete version of the Babel Interactive Web Interface that is pre-configured to trace FCG processes. The web interface is automatically initialised when the FCG Editor is launched and can be consulted at the address http://localhost:8008 using a web browser. The ‘Launch web interface’ button from the toolbar in the editor’s main interface can be used to automatically open a web browser at the right address. A screenshot of the web interface tracing the comprehension process of the utterance “*the linguist likes the mouse’*’ using a didactic demonstration grammar is shown in Fig 2. All elements of the visualisation are recursively expandable up to the level of the bare feature structures and unification bindings. The web interface can be cleared together with the output pane of the FCG Editor by clicking the button ‘Clear Output’ in the toolbar.

*Demo grammar*. Learning to use a new grammar formalism and development environment can be a challenging endeavour. To help novice users get acquainted with writing computational construction grammars, a didactic demonstration grammar fragment for English is available from the editor’s main interface. By clicking the button ‘Insert demo grammar’, the definition of a construction inventory with seven constructions, including a few lexical constructions, a noun-phrase construction and a transitive-clause construction, is added to the buffer that is currently open in the editor pane. This example grammar can help to learn the syntax of Fluid Construction Grammar, to learn to evaluate FCG constructs, to explore the web interface and output pane, and it can serve as basis for writing one’s own first grammar fragment.

*Comprehension and production from the GUI*. Once a construction inventory and its constructions have been evaluated, for instance by first clicking ‘New’, then ‘Insert demo grammar’, and then ‘Evaluate File’, the grammar is ready to be used for language processing. Given the bi-directionality of the FCG system, grammars can be used for both comprehending and producing utterances. Comprehension consists in mapping from an utterance to a representation of its meaning, while production performs a mapping from a meaning representation to an utterance that expresses it. For testing purposes, an utterance or meaning representation can be entered in the testing pane of the FCG Editor (see part E of Fig 1), along with the construction inventory to be used. Grammars are by default accessible via the global access point *fcg-constructions*. Utterances should be entered without any enclosing quotation marks unless these are part of the utterance, e.g. *the linguist likes the mouse*. Meaning representations should be specified in the form of predicates written in prefix notation, i.e. with an opening parenthesis before the predicate name rather than before the first argument, e.g. *(linguist x) (unique x) (mouse y) (unique y) (deep-affection x y)*. Upon hitting the Enter key or clicking the ‘Go!’ button in the testing pane, the FCG Editor automatically infers the required direction of processing based on whether the input is a sequence of characters or a sequence of predicates, and initiates the corresponding process (comprehension or production). The complete output becomes available in the interactive web interface and a summary of the result also appears in the output pane.

While the testing pane is envisioned as the primary way to test and explore new grammars, more specialised methods that are rooted in the FCG software library are also available within the editor. These methods involve function calls to ‘comprehend’, ‘comprehend-all’, ‘formulate’ and ‘formulate-all’, and can for example be included in loops for processing corpora rather than individual utterances. Also Babel’s parallel corpus processing package and testing and evaluation package can be accessed from within the FCG Editor.

*Grammar configurator*. Fluid Construction Grammar is an extensive toolbox that is highly customisable. It comes with a wide variety of pre-implemented processing and visualisation options that are accessible to the user through the specification of configuration settings in the definition of the construction inventory. These options affect the behaviour of the processing and visualisation engines, including many aspects of the definition of constructions, the search process involved in constructional language processing, meta-level operators that deal with unforeseen input, the hashing of constructions, and the visualisation of constructions and construction application processes. While suitable defaults are adopted by the FCG Editor to assist novice users in their learning process, grammar engineers working on larger projects soon want to be in full control of the behaviour of the constructional language processing system. A major challenge in this respect for the FCG user is to translate the desired behaviour into a combination of available configuration options, avoiding the holistic re-implementation of functionality that is already available through a combination of pre-implemented modules. In order to assist the user in this process, the FCG Editor features a helper tool in the form of a graphical grammar configurator.

The grammar configurator provides a graphical way to inspect and alter the configuration options of a construction inventory. Using expandable/collapsible elements, the user can navigate through all pre-implemented configuration options and visually inspect the configuration options that are currently set for the grammar under development. By clicking checkboxes, marking radiobuttons and filling text fields, the user can set different configurations in a straightforward manner. The interface guides the user in this process by providing all possible options and advising on dependencies between them. Certain options only appear if other options are selected, reflecting their compatibility and conditionality. The grammar configurator is an open-ended tool, in the sense that it provides at each point the possibility for the user to specify configuration options that they implemented themselves and are therefore not part of the standard FCG distribution.

**Fig 3:**
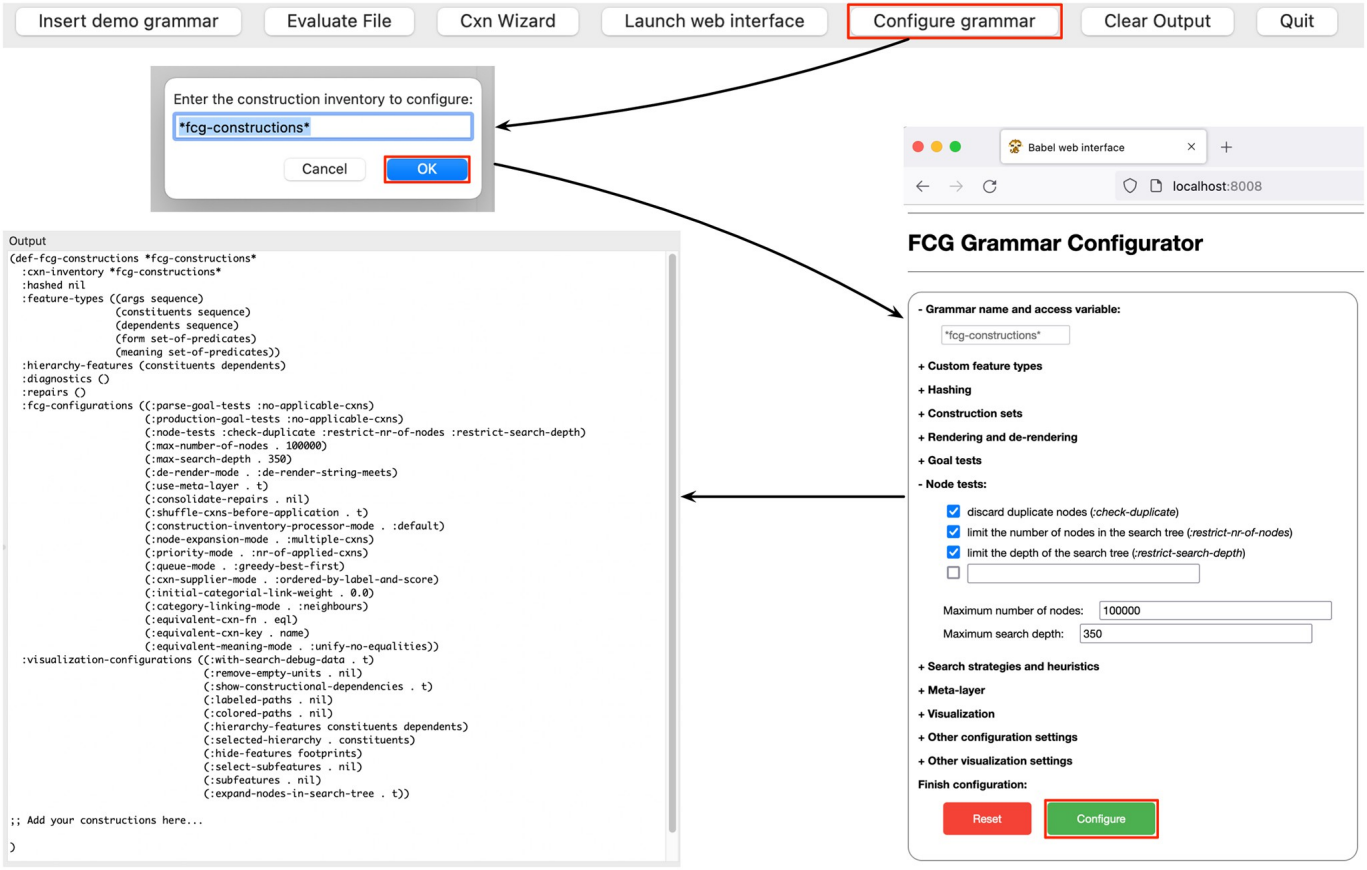
Grammar configurator. A graphical grammar configurator supports the user in fine-tuning the behaviour of the processing and visualisation engines.

The grammar configurator can be launched by clicking the ‘Configure grammar’ button in the toolbar. This opens a dialog window in which the user can specify the grammar to be configured. Upon clicking ‘OK’, the graphical grammar configurator appears in the FCG Editor’s web interface. After inspecting and altering the configuration options and subsequently clicking the ‘Configure’ button, the full configuration of the grammar is printed to the output pane of the FCG Editor. From there, it can be copied and pasted into the editor pane and integrated in the grammar. This process is visualised in Fig 3.

*Construction wizard*. When writing constructions, it is common practice to first design the skeleton of a construction, before implementing the features that capture its more intricate linguistic properties. The process of designing a construction skeleton is rather repetitive, time-consuming and error-prone. With the goal of supporting the grammar engineer in this task, the FCG Editor features a helper tool called *construction wizard*. The construction wizard automates the process of writing a construction skeleton based on a pattern of words provided by the user. The tool constructs the basic outline of a construction, adds units that match on the words present in the input pattern, and adds another unit that on the one hand matches on the pattern’s word order constraints and on the other hand groups all words together. Once added to the grammar, the skeleton built by the construction wizard is already an operational construction in itself, which can readily be used in the comprehension direction. Then, it is the task of the grammar engineer to further elaborate this construction by adding features that capture its meaningful linguistic properties, both on the form and on the meaning side of the construction.

**Fig 4:**
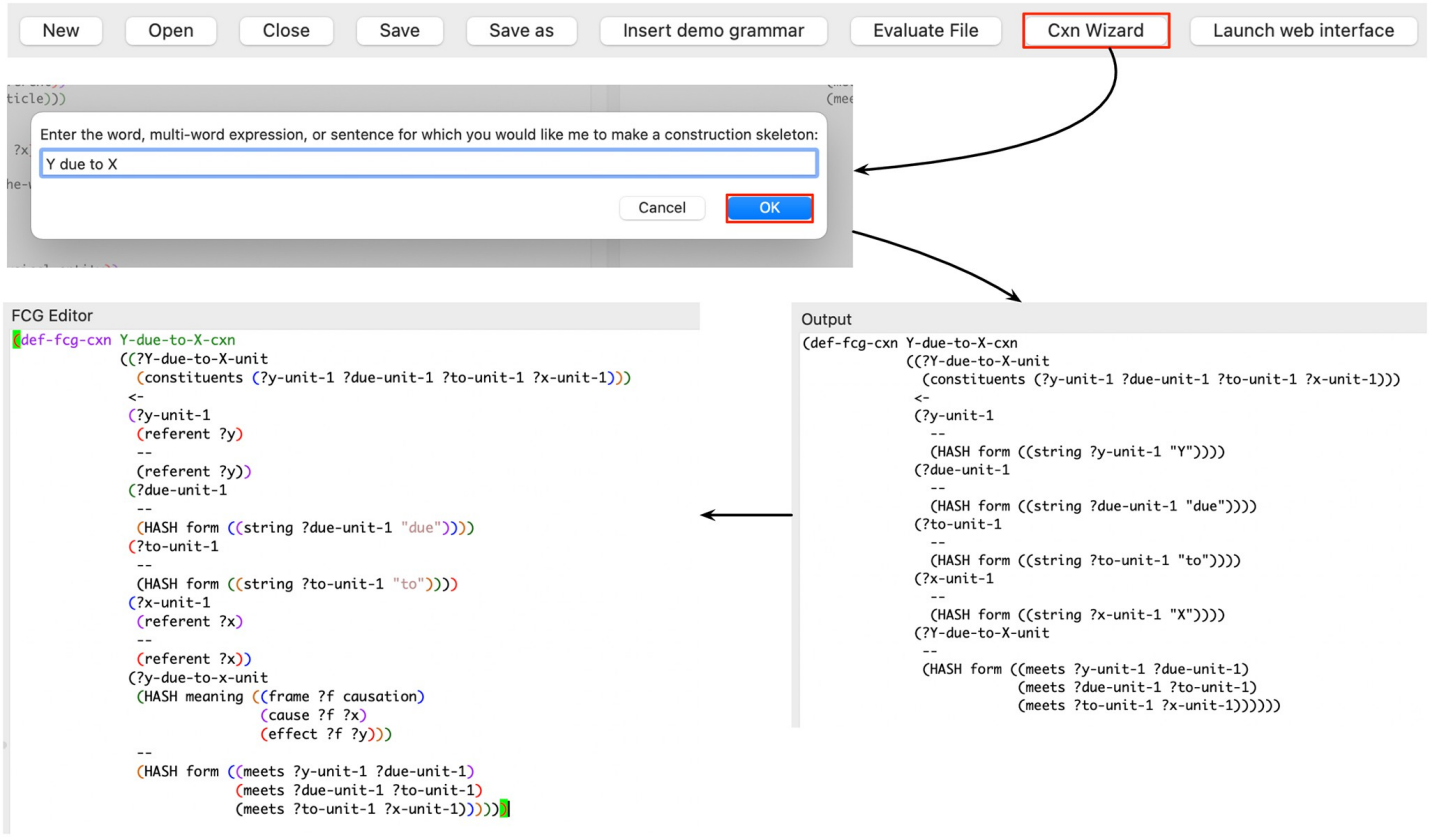
Construction wizard. After clicking the ‘Cxn Wizard’ button from the toolbar, a window opens in which the user can type a word, multi-word expression or sentence for which they wish to create a construction skeleton. Upon clicking ‘OK’, the construction skeleton is printed to the output pane, from where it can be copied to the grammar that is open in the editor pane. The grammar engineer can then further elaborate this construction and test it.

Fig 4 visualises the different steps involved in using the construction wizard. First, the construction wizard is called by clicking the ‘Cxn Wizard’ button in the toolbar of the FCG Editor. This causes a dialog window to open, asking the user to enter the pattern for which they would like to create a construction skeleton. After entering a pattern (in this case “*Y due to X*”) and clicking the ‘OK’ button, a skeleton for the Y-due-to-X-cxn appears in the FCG Editor’s output pane. This construction matches on the four elements of the pattern (“*Y*”, “*due*”, “*to*” and “*X*”), as well as on the ordering constraints between these elements. Additionally, the construction creates a new unit that groups together these four elements into a single unit. This skeleton can then be copied by the user to the editor pane, where they can build further on this skeleton to implement the exact construction they have mind. In the figure, this involves on the one hand specifying that the X and Y elements need a referent feature, and on the other hand adding features that capture the meaning side of the construction. In this case, the meaning is captured in the form of a causal frame, in which X fills the role of cause and Y fills the role of effect. Finally, the user can evaluate the construction to add it to the construction inventory, and test the resulting construction in both the comprehension and the production direction. This construction can contribute to the processing of utterances such as “*train traffic is disrupted due to a number of accidents*” and “*the school trip was postponed due to the Covid crisis*”.

*Managing running processes*. In an open-ended development environment like the FCG Editor, it is of crucial importance to be able to manage the processes that are running. For example, if a user starts the comprehension or production process of an utterance using an experimental grammar, it can happen that a combination of erroneous or non-optimal constructions causes the process to take much longer than expected. In this case, it is desirable to be able to kill the running process, adapt the grammar and restart evaluation. This prevents the situation where the user either needs to wait for the faulty process to finish or that faulty processes continue to run in the background slowing down the entire system.

The FCG Editor handles this challenge by running each evaluation event, for example triggered by hitting the Shift-Enter key combination or clicking the ‘Go!’ button in the testing pane, on a new thread. While this thread is running, a dialog window is shown. This dialog window informs the user that an evaluation process is in progress and offers the option to kill this process by clicking a ‘Cancel’ button. If the ‘Cancel’ button is not clicked, the window disappears once the evaluation process is finished. If it is clicked, the thread is destroyed immediately and the dialog window disappears. This solution ensures that the user always knows exactly which process is running, and that they can kill this process if desired.

*Debugging*. When implementing FCG grammars or Common Lisp source code, it is inevitable that errors will occur, for example due to violations of the required syntax. For the user experience, it is of great importance that errors are caught, and that insightful error messages and additional debugging information are displayed.

In the FCG Editor, each error during evaluation is caught on the editor level. The error message resulting from the evaluation process is displayed in a dialog window. The user then has two choices. The first option is to click an ‘Abort’ button, which kills the thread and makes the error message disappear. The second option is to click a ‘Report problem’ button, which kills the thread, makes the error message disappear and prints an extensive plain text error report to the output pane. The error report contains (i) the error message itself, (ii) the form that was being evaluated when the error occurred, (iii) a backtrace, (iv) information about the version of the editor, and (v) more low-level information including for example the packages that were loaded at the time of the error and the state of the garbage collector. As the extensive error report is printed to the output pane as plain text, it can easily be shared with the FCG user community or reported to the FCG development team.

*Source-level extensibility*. During the design of the editor, it was an explicit goal to cater to the needs of both regular users and FCG experts. FCG experts do not only need to be able to define new constructions and construction inventories, but also to be able to extend the FCG system with new features. For example, they might wish to add new ways to manage the search process involved in constructional language processing, write auxiliary functions to automatically build constructions based on dictionaries or other linguistic resources, or write functions that use their grammar for annotating corpora with constructional analyses. The combination of the advanced text editing functionalities, evaluation possibilities and debugging tools that are provided by the FCG Editor provide a powerful environment for extending the FCG system using Common Lisp source code.

*The FCG listener*. FCG grammars are typically developed in a prototyping style, where the exact specification is not known on beforehand. Constructions are implemented incrementally and tested continuously. During this spiral process, the grammar gradually grows, new challenges appear, and the grammar engineer designs solutions that improve the generality and coverage of the grammar. At the same time, the grammar engineer acquires linguistic insights that result from their interaction with the language processing engine. This style of development excellently fits the interactive programming paradigm, in which developers implement parts of a program while other parts are already operational.

**Fig 5:**
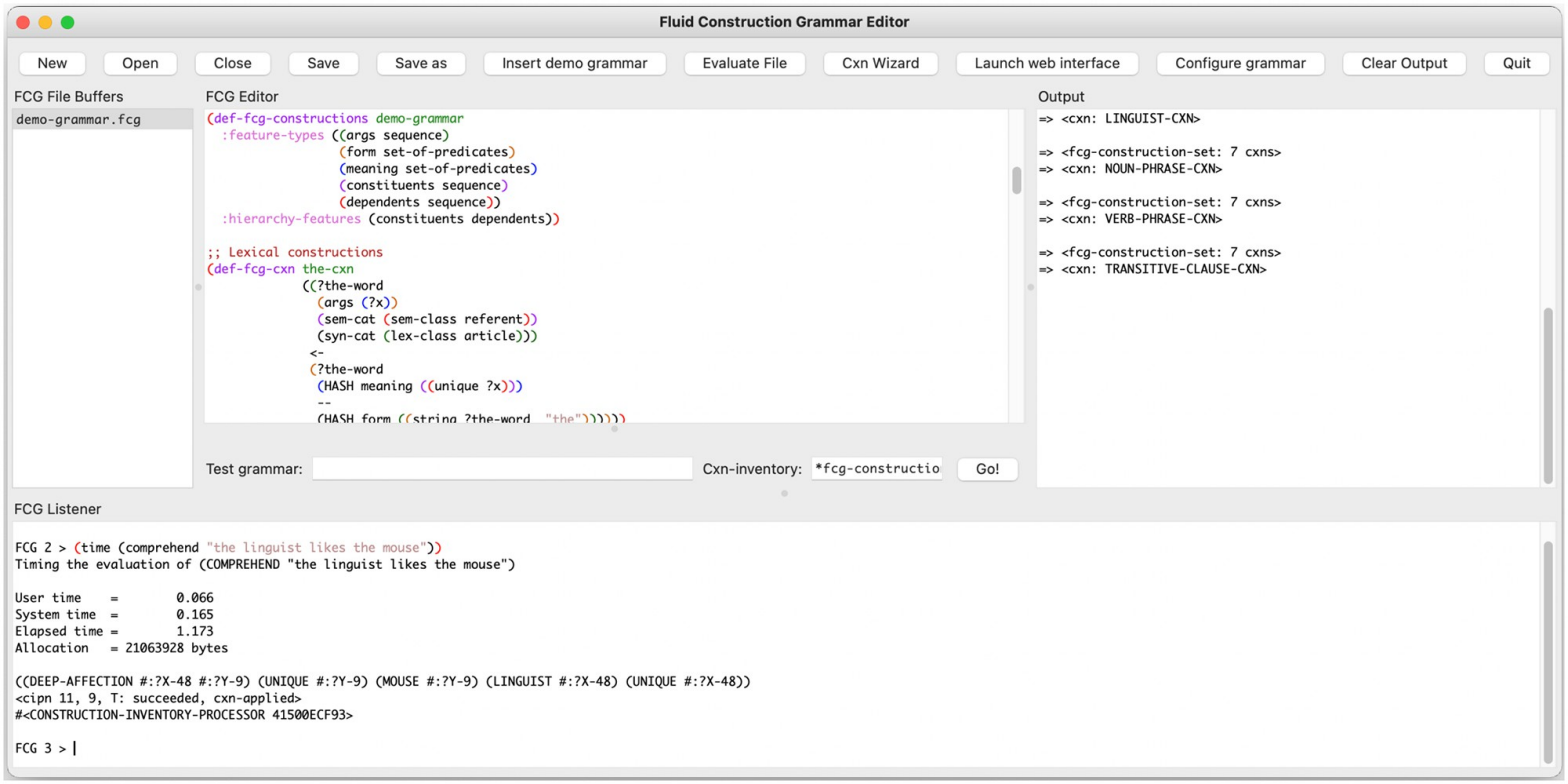
FCG listener. The FCG listener can be used to programmatically interact with a grammar, in this case timing the comprehension process of the utterance “*the linguist likes the mouse*” using the demo grammar.

The FCG Editor provides an interactive programming environment, in which the grammar engineer can interact with a grammar and its supporting source code during development. This interaction can happen on the one hand through the evaluation of FCG constructs, functions and function calls in the editor pane, and on the other hand using the FCG listener. The FCG listener provides a traditional Read-Eval-Print-Loop (REPL) that is connected to the running FCG system. This means that users can enter individual snippets of code that interact with the grammar. Upon entering these snippets, they are evaluated and the output is returned. This can for example be useful for quick testing, for timing the comprehension or production process of an utterance, or for manipulating grammars programmatically. Fig 5 shows how the FCG listener can be used to time the comprehension process of the utterance “*the linguist likes the mouse*” using the demo grammar.

#### Getting started

The FCG Editor can be downloaded for macOS, Microsoft Windows and Linux from the FCG user community website (https://www.fcg-net.org/download). The downloaded binary file can then be moved to the operating system’s application folder and launched like any other program, for example by double-clicking it. For visualising semantic networks in the web interface, one additional dependency needs to be installed, namely the Graphviz tool [113]. Instructions for doing so can be found on the FCG user community website.

Once the FCG Editor has been launched, the user can interact with it using its graphical user interface. A good start for novice users is to click the ‘New’ button for creating a new file, followed by clicking the ‘Insert demo grammar’ button that inserts a didactic demonstration grammar into the new file. The user can then click the ‘Evaluate File’ button and observe in the output pane how the construction inventory and each construction gets evaluated. Finally, the user can start interacting with the grammar using the testing pane, for example by entering utterances like “*the linguist likes the mouse*” or meaning representations like *(linguist x) (mouse y) (unique x) (unique y) (deep-affection x y)*. Upon clicking the ‘Go!’ button, the FCG engine starts comprehending or producing the utterance or meaning representation that was entered using the evaluated grammar. The output can be consulted at the address http://localhost:8008 using a web browser, and a summary of the result is printed to the output pane.

The user can then start to play around with the grammar, implement their own construction grammar insights and analyses, and contribute to the exciting methodological innovations that are currently taking place in the field of construction grammar.

#### Technical implementation

Technically, the FCG Editor connects a graphical user interface to an executable form of Babel’s Fluid Construction Grammar software library [104, 114]. The editor was built using LispWorks’ [115] delivery functionality and is distributed for macOS, Microsoft Windows and Linux in the form of an executable binary. The interface was built using LispWorks’ portable GUI toolkit CAPI (Common Application Programming Interface) [116], which provides a native look-and-feel on each operating system. The editor includes Lispworks’ Common Lisp interpreter, so that source code can be evaluated at runtime. All FCG source code is pre-loaded into the runtime image in its compiled form, and the input to the FCG listener, as well as the contents of all files created by the user, are automatically interpreted within the FCG-EDITOR namespace.

### Construction grammar engineering using the FCG Editor

The main motivation for the design and development of the FCG Editor was the combination of a growing interest in computational tools in the construction grammar community and a lack of accessible, user-friendly tools for operationalising computational construction grammars. Now that we have introduced the tool itself, we will in this section elaborate on how the tool aims to achieve its goal of supporting the computational exploration of novel construction grammar ideas on the one hand, and the large-scale engineering of construction grammars on the other.

#### Support for the exploration of novel construction grammar ideas

The suitability of the FCG Editor for exploring novel construction grammar ideas results from two main design choices. On the one hand, the tool integrates Fluid Construction Grammar as its underlying computational construction grammar formalism and constructional language processing system. This choice is motivated by the fact that Fluid Construction Grammar is not a linguistic theory in itself, but rather a special-purpose programming language that provides, but does not impose, high-level abstractions and building blocks for operationalising constructional language processing. On the other hand, the FCG Editor is designed as an open and extensible tool that supports the full range of possibilities offered by the FCG software library, and even supports extending many aspects of the FCG codebase. Support for the exploration of novel construction grammar ideas is achieved through the following properties:

**Support for partial analyses**. The constructional language processing engine is constructed in such a way that it explores the application of constructions given the input that is provided. This means that in principle, any construction application process leads to a resulting analysis to which zero or more constructions have contributed. For utterances or meaning representations that are only partly covered by the grammar, the resulting analysis still provides all information that was contributed by the constructions that could apply. Construction grammar engineers are typically not concerned with the traditional notion of grammaticality judgement. Grammars are evaluated in terms of the correctness of the mappings between form and meaning that result from their comprehension and production processes. The partial analysis of ungrammatical utterances, or the partial production of inconsistent meaning representations is considered an asset rather than a shortcoming.
**No feature or category declarations**. The features and categories that are used by the constructions of a grammar do not need to be defined outside the constructions themselves. There is no need to declare the values that a feature can take, nor does FCG impose any specific features to be used in a grammar. The use of features and categories is the responsibility of the grammar engineer alone, who should not feel constrained by the system when formalising their novel ideas.
**Free choice of meaning representation**. The FCG Editor does not impose a particular semantic theory or formalism to be used in a grammar, as long as meaning representations can be formalised as sets of predicates. Possible meaning representations thus include, but are not limited to, a variety of logic formalisms, variations on the lambda calculus, frame semantics, abstract meaning representation and procedural semantics.
**Word order constraints are optional**. In FCG, constructions can include word order constraints but they do not need to. For example, there is no need to include word order constraints in argument structure constructions. This is useful to explore grammars for languages where word order is driven by information structure rather than argument structure, for example in the case of languages that primarily rely on a case system for expressing argument structure.
**Non-contiguous phrases**. Constructions affecting linguistic units that are non-contiguous are not special in any way. They can for example include word order constraints between specific units only, in terms of adjacency or precedence, or leave out word order constraints altogether.
**Non-locality of constructions**. Constructions are non-local in the sense that they can access all linguistic information that is known at a certain point in processing. This includes both information that was part of the input utterance or meaning representation, and information that was contributed by other construction applications.
**Constructions do not necessarily build trees**. Constructions contribute linguistic information that can subsequently be used by other constructions. They do not necessarily correspond to tree-building operations [95]. If desired by the grammar engineer, the feature structures that are used can be considered to represent trees. This can be useful for reasons of information structuring and visualisation. However, constructions can also build multiple different tree or network structures at the same time, or even not incorporate any notion of tree-building at all.
**Support for procedural attachment**. Construction application is normally performed by unification operations [35, 117]. However, it can sometimes be desired to perform other kinds of computation on features or their values. For example, one might want to compute the ontological distance between categories using numerical or graph-distance operations during construction application. This is possible in the FCG Editor through the use of an expansion operator, which allows the user to extend FCG’s unification operations with dedicated procedures for chosen features or their values.
**Modelling of entrenchment patterns**. The FCG Editor facilitates the modelling of entrenchment patterns by assigning scores to constructions and making use of these scores to steer the precedence of construction applications during the search process that constitutes constructional language processing [99, 118]. These entrenchment scores can be set by the grammar engineer, or they can be automatically updated based on their occurrence in corpora of language use. This facilitates the ranking of analyses in terms of their entrenchment in a usage-based fashion.

In sum, the FCG Editor provides a number of ready-to-use building blocks that operationalise the basic tenets of construction grammar, while supporting the freedom of the grammar engineer to explore novel construction grammar ideas, insights and analyses. Thanks to its straightforward installation and user-friendly graphical interface, even construction grammarians without extensive programming experience can benefit from the advantages of including computational modelling into their research.

#### Support for large-scale construction grammar engineering

The suitability of the FCG Editor for large-scale construction grammar engineering primarily stems from the combination of a user-friendly interface with a performant Fluid Construction Grammar-based backbone. The FCG Editor thereby provides a variety of building blocks that support the grammar engineer in designing, implementing and processing large grammars:

**Efficient processing**. Efficient constructional language processing is provided by the Fluid Construction Grammar-based backbone of the FCG Editor. Using common optimisation techniques such as hashing of constructions and heuristic search strategies, it can efficiently process grammars of over 100,000 constructions. Using the built-in corpus processing package, it supports the parallel processing of text corpora on multi-core machines.
**Evaluation and regression testing**. Evaluation and regression testing is provided by the built-in grammar evaluation package [83], which reports on timing, accuracy, coverage, and quantitative aspects of the explored search space. Accuracy of analyses is quantified in terms of smatch-like scores for meaning representations [119] and edit distance metrics for utterances.
**Meta-level processing**. FCG integrates a meta-level architecture that separates routine processing from the meta-level processing of unforeseen input [120]. This architecture constantly monitors the construction application process, diagnoses problems, and applies repair strategies where applicable. Most commonly, repair strategies are used to learn new lexical constructions during processing or to generalise and specialise existing constructions with respect to novel input [109].
**Automation of the grammar engineering process**. The FCG Editor provides a number of helper tools that automatise part of the construction grammar engineering process. The construction wizard automatises the time-consuming and error-prone process of writing down the basic structure of constructions. Not only does this speed up the grammar development process, it also eliminates syntax errors and common bugs resulting from inconsistencies in the naming of units, variables, subunits and ordering constraints. The grammar configurator helps users configure their grammars by automatically generating configuration code based on a user-friendly form that consists of a combination of checkboxes, radiobuttons and textfields.
**Visualisation and debugging**. FCG’s interactive web interface provides an orderly yet detailed visualisation of all aspects involved in the process of comprehending or producing utterances, including the search process involved and the unification bindings for individual construction applications. The web interface also includes an anti-unification-based diagnostic tool that determines which features or variable bindings block the application of a given construction [109].

## Discussion and conclusion

In this paper, we have introduced the FCG Editor as a user-friendly, feature-rich and open-ended tool for implementing computational construction grammars. The design and development of the tool were primarily motivated by the rapidly growing importance of computational modelling within the field of construction grammar. In particular, the FCG Editor caters to the needs of construction grammarians who wish to automatically verify the consistency and preciseness of their analyses, corroborate their analyses with corpus data, or use their construction grammar insights and analyses for enhancing the performance of language technology applications.

The requirements analysis that was carried out in concertation with members of the construction grammar community in the run-up to the development of the FCG Editor brought to light three important design aspects. First of all, the tool needed to be user-friendly for novice users with no extensive programming experience. At the same time, the tool needed to be open-ended and extensible, so that expert users would feel no restrictions when exploring novel, outside-the-box ideas. Finally, the tool needed to be interactive on all levels, so that users would get a detailed insight into all aspects of constructional language processing, including the intricate interactions between the constructions of a grammar.

The first concrete requirement was that the editor should offer the basic text formatting functionalities that are commonly featured in text editors intended for programming (R1). This has been operationalised in the FCG Editor by including basic functionalities for manipulating files and editing text, supplemented by syntax highlighting for both FCG and Common Lisp syntax, auto-completion, auto-indentation, block comment formatting, and the display of function arguments. The look-and-feel of the editor is native on each operating system, and its key bindings are designed to reflect those used in other modern programming environments. The second requirement was that the editor should offer a straightforward and cross-platform installation, and that external dependencies should be limited to the bare minimum (R2). The FCG Editor is available for Linux, macOS and Microsoft Windows, and can be installed in a single click. For an optimal user experience, one external dependency needs to be installed, namely the Graphviz visualisation tool which is freely available for the three operating systems. The third requirement was that the editor should make implementing computational construction grammars more accessible to novice users (R3). This is achieved by including a manual, a didactic example grammar, a construction wizard, a grammar configurator, and a testing pane for comprehending and producing utterances from within the editor’s main interface. The fourth requirement was that the editor should include visualisations for constructions and construction application processes, so that all details can be graphically inspected by the user (R4). The FCG Editor provides this functionality through the integration of a version of Babel’s interactive web interface that is pre-configured to trace FCG processes. The fifth requirement was that the editor should include facilities for catching errors and displaying insightful error messages (R5). The FCG Editor satisfies this requirement by catching all errors that occur during evaluation and by providing the possibility to print elaborate reports about these errors in plain text. The sixth requirement was that users should be able to manage running processes in an intuitive way (R6). This is achieved by dispatching each evaluation process to a separate thread, and providing the possibility to kill these threads by clicking a ‘Cancel’ button. The seventh requirement was that the editor should offer the possibility to write source code that extends the FCG system (R7). This requirement is satisfied by including a Common Lisp interpreter in the FCG Editor and providing the possibility to evaluate blocks of code. The final requirement was to provide an interactive programming environment through which users can interact with their grammar and the novel code they write (R8). This requirement is met through the inclusion of a REPL-style listener in the editor’s GUI, which allows advanced users to programmatically interact with their grammar and code.

Functionally, the FCG Editor supports two main types of usage. First, it assists the construction grammarian in computationally exploring novel ideas, thereby offering a more solid methodological foundation for theory building within the field of construction grammar. It does this by providing accessible computational operationalisations of the basic tenets of construction grammar, while imposing as few theoretical assumptions as possible. The FCG Editor’s back-end supports non-local constructions, constructions without word order constraints, constructions that do not correspond to tree-building operations and constructions that handle non-contiguous patterns. It offers a free choice when it comes to the format used for representing meaning, does not require the grammar engineer to centrally define possible features and categories, and provides support for procedural attachment within constructions. The system also supports the modelling of observed language use, by offering the possibility to formalise entrenchment patterns on the one hand, and to handle unforeseen input by means of partial analyses and meta-level repair operations on the other. The second type of usage supported by the FCG Editor concerns the large-scale engineering of computational construction grammars. This is achieved through the integration of efficient constructional language processing algorithms and optimisation strategies, support for evaluation and regression testing, helper tools that automatise part of the grammar engineering process, and an extensive interactive web visualisation system that can be used for debugging and optimisation purposes.

A central objective of the research programme that has led to the development of the FCG Editor consists in developing novel techniques and tools that support researchers in the field of construction grammar in transforming their theories and analyses into fully operational computational models. Faithful computational operationalisations of construction grammar bring important methodological advantages that are expected to have a major impact on the field, especially when it comes to the challenge of scaling constructionist approaches to language. First of all, computational operationalisations are crucial for automatically validating the preciseness and internal consistency of construction grammar theories and analyses, as it is impossible to do this by hand for large-scale grammars. Second, computational operationalisations make it possible to corroborate construction grammar theories and analyses with large amounts of corpus data, thereby scaling the usage-based aspects of construction grammar research. Third, computational operationalisations allow moving away from studying individual constructions to studying the systemic relations between families of constructions. The possibility to model these intricate relations on a large scale is of crucial importance for scaling construction grammar theories beyond the current state of the art. Finally, computational operationalisations of construction grammar can help to standardise the way in which constructions are represented, thereby facilitating the exchange of ideas and results among researchers.
